# Using large biobanks for psychiatric genomic research: Consistency of clinical and genetic aspects of recorded depression across US states in the All of Us Research Program

**DOI:** 10.1017/S0033291725102420

**Published:** 2026-01-23

**Authors:** Katherine M. Keyes, Catherine Gimbrone, Caroline Rutherford, Yingzhe Zhang, Karmel Choi, Louisa Smith, Philip Greenland, Jordan W. Smoller, Maria Argos

**Affiliations:** 1 Department of Epidemiology, Columbia University Department of Epidemiology, New York, USA; 2 Columbia University Department of Epidemiology, USA; 3 Columbia University, USA; 4 Harvard University Department of Epidemiology, USA; 5 Massachusetts General Hospital Department of Psychiatry, USA; 6 Northeastern University Bouve College of Health Sciences, USA; 7 Northwestern University Feinberg School of Medicine, USA; 8 Boston University, USA

**Keywords:** biobank, Depression, Epidemiology, genetics, generalizability, Polygenic risk score

## Abstract

**Background.:**

Large biobanks offer unprecedented data for psychiatric genomic research, but concerns exist about representativeness and generalizability. This study examined depression prevalence and polygenic risk score (PRS) associations in the *All of Us* data to assess potential impacts of nonrepresentative sampling.

**Methods.:**

Depression prevalence and correlates were analyzed in two subsamples: those with self-reported personal medical history (PMH) data (N = 185,232 overall; N = 114,739 with genetic data) and those with electronic health record (EHR) data (N = 287,015 overall; N = 206,175 with genetic data). PRS weights were estimated across ancestry groups. Associations of PRS with depression were examined by state and ancestry.

**Results.:**

Depression prevalence varied across states in both PMH (16.7–35.9%) and EHR (0.2–45.8%) data. Concordance between PMH and EHR diagnoses was low (kappa: 0.29, 95% CI: 0.30–0.30). Overall, one standard deviation increase in depression PRS was associated with lifetime depression based on PMH (odds ratio [OR] = 1.05, 95% confidence interval [CI]: 1.04–1.07) and EHR (OR = 1.05, 95% CI: 1.04–1.07). Results were generally consistent by ancestry, with the strongest signal for European ancestry (PMH: OR = 1.10, 95% CI: 1.08–1.12; EHR: OR = 1.07, 95% CI: 1.05–1.10). Associations between PRS and lifetime depression were largely consistent and significant associations varied minimally (ORs = 1.06–1.45) by state of residence in both subsamples.

**Conclusions.:**

Recorded depression prevalence by state in *All of Us* demonstrates a wide range, likely reflecting recruitment differences, EHR data completeness, and true geographic variation; yet PRS associations remained relatively stable. As studies like *All of Us* expand, accounting for sample composition and measurement approaches will be crucial for generating actionable findings.

## Introduction

Major depression remains a leading determinant of health and longevity globally (GBD 2019 Mental Disorders Collaborators, 2022). An increasing prevalence of depression and its sequelae, including suicide, in the United States and elsewhere (Keyes & Platt, 2024; Martínez-Alés, Jiang, Keyes, & Gradus, 2022) signals the continued need to invest in identifying causes and determinants, including the complex interplay of genetic factors, modifiable environmental exposures, and social determinants at a population level.

Large biobanks, with extensive data on genomics and other -omics, health and healthcare utilization, and social and environmental exposure data, are rapidly expanding across many countries, generating extraordinary scientific output, including for understanding psychiatric disorders. Among the most recently initiated of these large biobanks is the *All of Us* Research Program (Denny et al., 2019), which has enrolled over 849,000 individuals residing in the United States as of March 2025 (Data Snapshots, 2025). The *All of Us* Research Program has placed mental health research among the priority areas of its scientific roadmap (All of Us Research Program, 2023). Ensuring that the science generated from large biobanks is relevant to the populations for which we will use the science to intervene is paramount, given the breadth and depth of participant data collection and involvement, expense, and potential promise.

A concern across biobanks, including *All of Us*, has been the representativeness of the populations from which they are drawn (Fry et al., 2017; Keyes & Westreich, 2019; Zeng et al., 2024) and potential selection bias compared to the underlying source population (Bradley & Nichols, 2022; Fry et al., 2017; Lee et al., 2023; Schoeler et al., 2023). Studies of the UK Biobank, for example, have demonstrated that those who participate are generally healthier than the general population (Batty, Gale, Kivimäki, Deary, & Bell, 2020; Stamatakis et al., 2021; van Alten, Domingue, Faul, Galama, & Marees, 2024) and that participation in biobank studies may itself have genetic determinants (Benonisdottir & Kong, 2023; Schoeler et al., 2023). In *All of Us*, participants are generally older, more racially diverse, and have higher income than the general population of the United States, and with a higher prevalence of major chronic diseases (Kathiresan et al., 2023; The All of Us Research Program Genomics Investigators, 2024; Zeng et al., 2024). This is not unexpected, as participants were largely recruited from medical centers across the United States, with a mission to enroll participants historically underrepresented in biomedical research (All of Us Research Program, 2024b). Furthermore, enrollment sites differ in their engagement and recruitment practices for the *All of Us* Research Program, which may yield greater participation among individuals from certain communities or with specific disease conditions. While benefits of these recruitment approaches include that data are enriched for health outcomes of interest, which allows greater statistical power, bias can and does arise that may mitigate the validity of results even in the face of greater statistical power. For example, bias can arise if factors associated with participation in the study interact with exposures of interest (Keyes, Smith, Koenen, & Galea, 2015) or are deterministic of both independent and dependent variables of interest (causing collider bias) (Munafò, Tilling, Taylor, Evans, & Davey Smith, 2018). These concerns may be amplified in data in which linkages to electronic health records (EHRs) are voluntary or have other selection criteria. The extent to which misalignment between the *All of Us* cohort and the U.S. population may generate science that is not broadly applicable remains understudied.

The present study has three aims to explore the potential consequences of nonrepresentativeness and variable outcome ascertainment for depression research. First, we examine the prevalence of recorded depression by mode of data collection (self-reported medical histories versus EHRs). Second, we examine the correlates of available genomic data in the *All of Us* Research Program to ascertain the potential for selection and nonrepresentativeness within key *All of Us* subsamples. Third, within the sample with genomic data, we examine the association between PRS for depression based on the largest available meta-analyses (Major Depressive Disorder Working Group of the Psychiatric Genomics Consortium, 2025; Meng et al., 2024) with ascertained depression status in *All of Us*, by genetic ancestry and participant U.S. state of residence. We estimate the associations of PRS with depression across U.S. states to test for potential variability based on factors associated with participation, given that sites across states recruited in unique ways. In doing so, we highlight possible strengths and limitations of inference from genetic studies using *All of Us* associated with nonrepresentative sampling.

## Methods

### Data source and sample

Data were drawn from the *All of Us* Research Program. Participation in *All of Us* is open to volunteers aged 18 years and older, and recruitment has occurred primarily through healthcare provider organizations throughout the United States (Denny et al., 2019). Once consented, eligible participants are invited to complete baseline surveys (e.g. ‘personal medical history’ [PMH]), provide authorization to link EHR data, undergo assessment of physical measures, and contribute to biological specimens. After the baseline assessment, participants are invited to complete additional health surveys. Additional details of the study protocol can be found elsewhere (Denny et al., 2019). The present study focused on two subsamples of *All of Us* participants: those who completed the baseline PMH module (‘PMH sample’) (N = 185,232) and those who authorized EHR linkage (‘EHR sample’) (N = 287,015).

### Measures

#### Polygenic risk score (PRS) for depression

The PRS weights were generated using PRS-CSx (Ruan et al., 2022), a Bayesian method that integrates genetic effects across populations through a shared continuous shrinkage (CS) prior, resulting in more precise effect size estimates. We calculated these weights using the most recent depression genome-wide association study summary statistics (Major Depressive Disorder Working Group of the Psychiatric Genomics Consortium, 2025; Meng et al., 2024) from multiple ancestry groups: African, Admixed American, East Asian, European, and South Asian. Then, the PRS for depression was calculated using PLINK2 for each individual based on the transancestry weights from PRS-CSx. Quality control was performed on variants from the HapMap 3 reference panel extracted from whole genome sequencing data by removing related samples and excluding flagged samples and variants from the All of Us v7 genomic dataset (The All of Us Research Program Genomics Investigators, 2024). Specifically, we filtered out variants with a minor allele count <1 or Hardy–Weinberg equilibrium P < 1 × 10^−10^ among multiancestry HapMap3 variants within each ancestry (The All of Us Research Program Genomics Investigators, 2024).

#### Genetic ancestry

Genetically identified ancestry was coded in the All of Us (AoU) sample based on a random forest classifier model (The All of Us Research Program Genomics Investigators, 2024). The ancestry categories follow the same labels from multiple sources including gnomAD, the Human Genome Diversity Project, and 1,000 Genomes. Categories in our analyses included African, Admixed American, East Asian, European, and South Asian. Middle Eastern population was not included due to insufficient sample size and the absence of corresponding summary statistics.

#### Self-reported lifetime depression in the PMH data

In the PMH data, lifetime depression was measured by two items harmonized collected in the baseline survey. Participants were coded as having lifetime depression if they answered positively to ‘Has a doctor or health care provider ever told you that you have depression?’ in V6 and earlier surveys or if they answered ‘self’ to the question: ‘Including yourself, who in your family has had depression? Select all that apply’ in V7 surveys. To remain consistent with DSM hierarchical criteria, those who reported lifetime bipolar disorder or schizophrenia were not coded as having lifetime depression even if they answered ‘yes’ or ‘self’ to the questions above.

#### Diagnosed lifetime depression in the EHR data

Participants provided separate consent at enrollment to share EHR data with the program. Currently, most EHRs available in the *All of Us* dataset are provided by enrollment centers, which are the medical centers where a participant receives healthcare. We used the eMERGE diagnostic coding schema to capture lifetime depression for those with EHR data (Carrell, 2018; McCarty et al., 2011), adhering to the eMERGE 2/30/180 rule, which requires qualifying diagnostic codes to be present on at least two distinct calendar days that are at least 30 days apart and not more than 180 days apart. Participants were coded as having lifetime depression based on ICD-9 and ICD-10 diagnostic codes indicative of major depression (see Supplementary Table S1 for all codes used). Those with bipolar disorder or nonmajor depression identified via ICD diagnostic codes were not coded as having depression for our main analyses. As a sensitivity analysis, we expanded the outcome to include other diagnostic codes for ‘nonmajor depression’ (see Supplementary Table S1). Depression criteria aligned with definitions of lifetime depression in the PRS meta-analyses, including self-reported history of depression, and medical records of depression diagnosis.

#### Demographics

Some demographic variables were recoded to mask sample sizes <20 as required by the *All of Us* Data and Statistics Dissemination Policy (All of Us Research Program, 2024a) and delineated below. We used demographics characteristics from modules self-reported by participants; included were categorical age (at the time of the baseline survey for the EHR sample, and at the time of the first PMH survey administration for the PMH sample, categorized as <25, 25–54, and >54), gender identity (other category combined transgender, nonbinary, and additional options), race/ethnicity (Asian combined with Native Hawaiian or Pacific Islander), highest educational attainment, categorical annual income (<$25,000, $25–50,000, $50–100,000, and > $100,000), and survey language. We also included self-reported state of residence at the time of enrollment.

#### Statistical analyses

Chi-squared tests with continuity correction were used to assess group differences among samples stratified by demographic variables. Cohen’s kappa (McHugh, 2012) was used to assess the concordance between lifetime self-reported depression in the PMH samples and lifetime depression in the EHR samples for those with overlapping data. Logistic regression was used to estimate odds ratios and corresponding 95% confidence intervals (CIs) for the relationship between a one standard deviation change in PRS and depression outcomes (PMH reported considered separately from EHR recorded) in overall samples as well as in samples stratified by genetic ancestry and state of residence. State of residence was included as a stratification variable only for those analyses in which there were greater than 500 respondents per state, given statistical power considerations. False discovery rate (FDR) corrections were applied to logistic regression analysis p values (Benjamini & Hochberg, 1995). Models additionally adjusted for population structure using the genetic principal components (PCs) with 10 PCs controlled in regression models. Models with sample sizes <500 overall and/or ≤5 for either response to a binary depression outcome were excluded from analyses in order to ensure statistical power and compliance with data dissemination policies (All of Us Research Program, 2024a). All analyses were conducted within the *All of Us* Researcher Workbench using version V7 of the *All of Us* data. Analyses were completed using R version 4.4.0 and Hail version 0.2.130 for Python to extract genomic data.

## Results

### Sample characteristics

The present study focused on two subsamples of *All of Us* participants: those who completed the baseline PMH module (‘PMH sample’) (N = 185,232) and those who had available EHR data (‘EHR sample’) (N = 287,015). Supplementary Figure S1 is a flowchart outlining participants included in these two subsamples for this analysis. Within the PMH and EHR samples, all participants were included in the descriptive analyses, but state-level analyses were conducted if there were 500 participants with EHR/PMH data within a U.S. state, resulting in 105,296 participants in state-specific descriptive analyses of the PMH sample and 189,664 participants in state-specific descriptive analyses of the EHR sample. The association between PRS and depression was assessed among participants with available genomic data. The genomic subsamples were further pruned based on kinship scores of 0.1 or greater and restricted to those with PRS data. This resulted in 108,928 participants in the PMH genomic subsample and 192,667 participants in the EHR genomic sample. Some participants contributed both PMH and EHR data such that the samples included an overlap of 131,392 individuals, and the genomic subsamples included an overlap of 94,848 individuals.

### Prevalence of recorded depression and concordance across data sources

**Figure 1.**
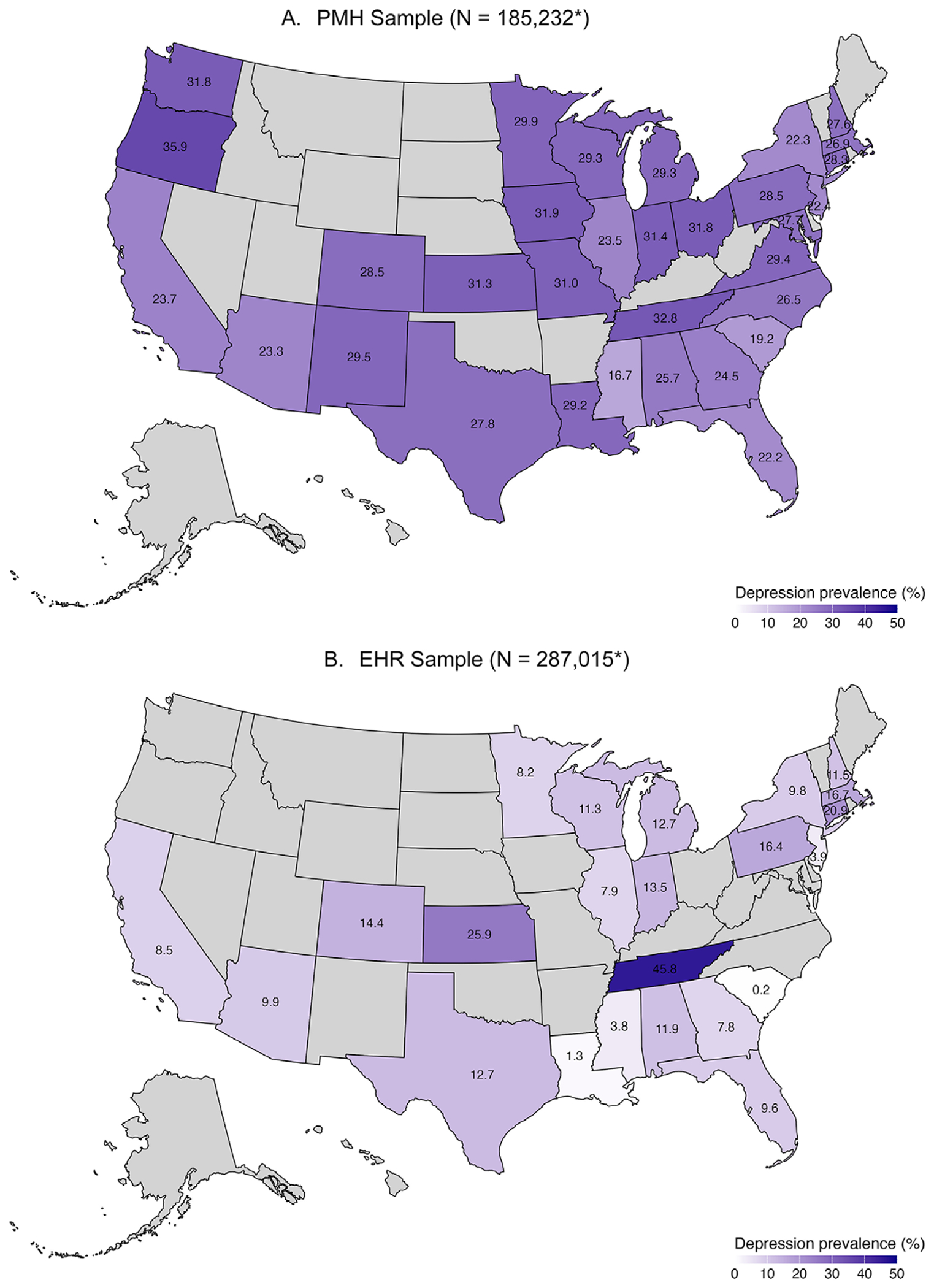
Ascertained depression prevalence by sample* and state of residence. (A) PMH Sample (N = 185,232*). (B) EHR Sample (N = 287,015*). *In the PMH sample, data are based on self-reported depression. In the EHR sample, data are based on available EHR records. Note that not all sites provided access to mental health EHR data; thus, the prevalence is based on what was submitted and may not reflect total depression prevalence in all possible EHR data. *Note:* Participants in states in gray were excluded from analyses if the state did not enroll any participants, or if the number of enrolled participants was less than 500. States included in analyses: Alabama, Arizona, California, Colorado, Connecticut, Florida, Georgia, Iowa, Illinois, Indiana, Kansas, Louisiana, Massachusetts, Maryland, Michigan, Minnesota, Missouri, Mississippi, North Carolina, New Hampshire, New Jersey, New Mexico, New York, Ohio, Oregon, Pennsylvania, South Carolina, Tennessee, Texas, Virginia, Washington, and Wisconsin.

Figure 1 shows the prevalence of ascertained major depression in the *All of Us* cohort by the U.S. states. In the PMH sample, lifetime self-reported depression prevalence ranged from 16.68% in Mississippi to 35.94% in Oregon. In the EHR sample, the lowest recorded depression prevalence was 0.20% in South Carolina. Tennessee was an outlier among those states with available EHR data, with a prevalence of 45.80%.

Concordance between self-reported depression and diagnosed depression using the eMERGE diagnostic coding scheme was low between PMH and EHR data for those participants with both data sources (kappa: 0.30, 95% CI: 0.29–0.30). There was also low concordance when comparing self-reported depression with the expanded measure of depression that additionally included nonmajor depression diagnostic codes used in sensitivity analyses in the EHR subsample (kappa: 0.33, 95% CI: 0.32–0.33). Findings were similar for both the primary and genomic subsamples. Supplementary Table S2 shows kappa values for the concordance of depression between samples stratified by state of residence. Concordance between self-reported depression and EHR diagnosed depression ranged from 0 (95% CI: −0.02 to 0.02) in South Carolina to 0.37 (95% CI: 0.33–0.40) in Florida. States with kappa values close to 0 likely reflect those with incomplete data linkage to mental health visits.

### Demographic correlates of recorded depression by data source

The demographic distributions for lifetime depression in the PMH and EHR samples are shown in Table 1. Diagnosed lifetime depression prevalence was lower in the EHR sample (11.3%) compared to self-reported lifetime depression prevalence in the PMH sample (26.2%). Notable differences in correlates emerged. In both the PMH and EHR samples, higher depression prevalence was associated with female and other gender identity, White and multipopulation racial identification, and lower annual reported income. In both samples, the prevalence of depression was lower among those with available genetic data versus those without. In contrast, higher depression prevalence was associated with younger age in the PMH sample and older age in the EHR sample, as well as higher educational attainment in the PMH sample but no association between educational attainment and recorded depression in the EHR sample. Participants completing surveys in Spanish had substantially lower prevalence of self-reported depression in the PMH sample, but survey language preference was not associated with diagnosed depression in the EHR sample. Supplementary Table S3 presents the demographic distributions in the EHR sample for the expanded measure of depression that additionally included nonmajor depression diagnostic codes used in sensitivity analyses. Findings were similar to those for the primary definition of diagnosed depression.

Supplementary Table S4 shows the demographic characteristics of the PMH and the EHR samples, stratified by whether the participants have available genetic data to date. In the PMH sample, availability of genetic data was positively associated with older age, Hispanic/Latino ethnicity, and Spanish survey language; those reporting ‘other’ gender identity were less likely to have available genetic data. Additionally, those with self-reported lifetime depression were also less likely to have available genetic data. In the EHR sample, older participants were also more likely to have available genetic data, but other demographic correlates differed; the availability of genetic data was associated with self-identified White race, higher income, and education attainment, while survey language and gender identity were not associated.

### PRS associations with recorded depression by data source and the U.S. state

**Figure 2.**
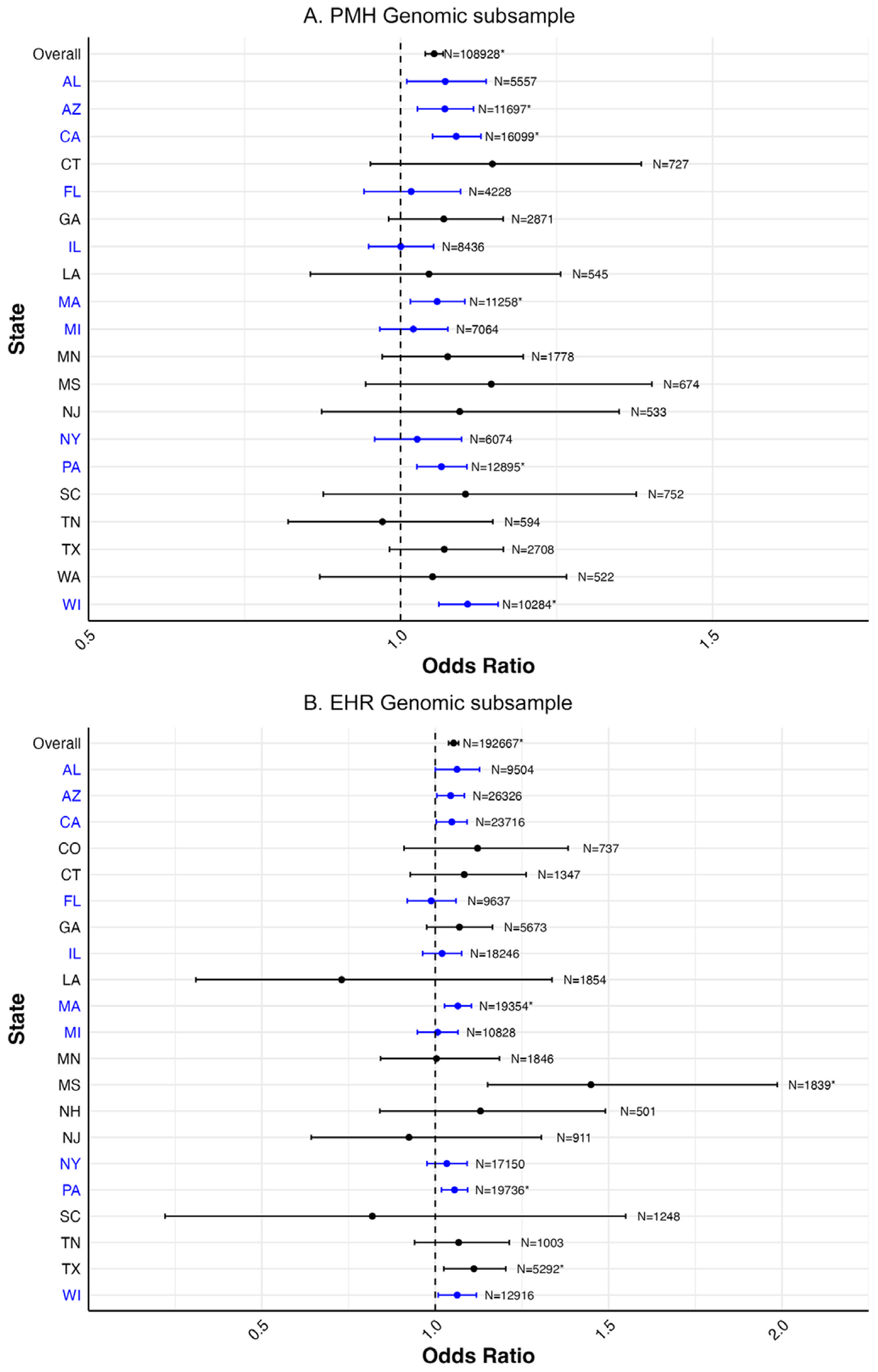
Forest plot of odds ratios and 95% confidence intervals for one standard deviation change in PRS with lifetime depression overall and stratified by state of residence in both the PMH (N = 108,928) and EHR (N = 192,667) genomic subsamples. (A) PMH genomic subsample. (B) EHR genomic subsample. *Notes:* States and corresponding estimates in blue denote locations of *All of Us* enrollment centers (All of Us Research Program, 2024b). Models adjusted for 10 PCs. *Statistical significance after false discovery rate correction.

In the PMH sample (Figure 2A), overall, a one standard deviation higher PRS was associated with higher odds of self-reported lifetime depression (OR: 1.05, 95% CI: 1.04–1.07), in models adjusted for 10 PCs. In analyses stratified by state of residence, there was appreciable variation in the observed odds ratio across states, with the most variation in the states with the smallest sample sizes. After FDR correction, the PRS was significantly associated with self-reported lifetime depression in five out of the 20 states evaluated, with odds ratios for those six states ranging from 1.06 (95% CI: 1.02–1.10) to 1.11 (95% CI: 1.06–1.16). The specific odds ratios and 95% CIs underlying Figure 2A are provided in Supplementary Table S5.

In the EHR sample (Figure 2B), overall, a one standard deviation higher PRS was associated with a higher odds of diagnosed depression recorded in the EHR (OR: 1.05, 95% CI: 1.04–1.07), in models adjusted for 10 PCs. Of note, the magnitude and precision of the odds ratio and the 95% CIs were essentially identical between the PMH and EHR samples. Similar to the PMH sample, variation in the odds ratio magnitudes and CI widths were mostly aligned with sample size. After FDR correction, there was a statistically significant association in four out of the 21 states evaluated, with odds ratios ranging from 1.06 (95% CI: 1.02–1.09) to 1.45 (95% CI: 1.15–1.99). The specific odds ratios and 95% CIs underlying Figure 2B are provided in Supplementary Table S5.

### PRS associations with recorded depression by data source and the U.S. state, stratified by genetic ancestry

**Figure 3.**
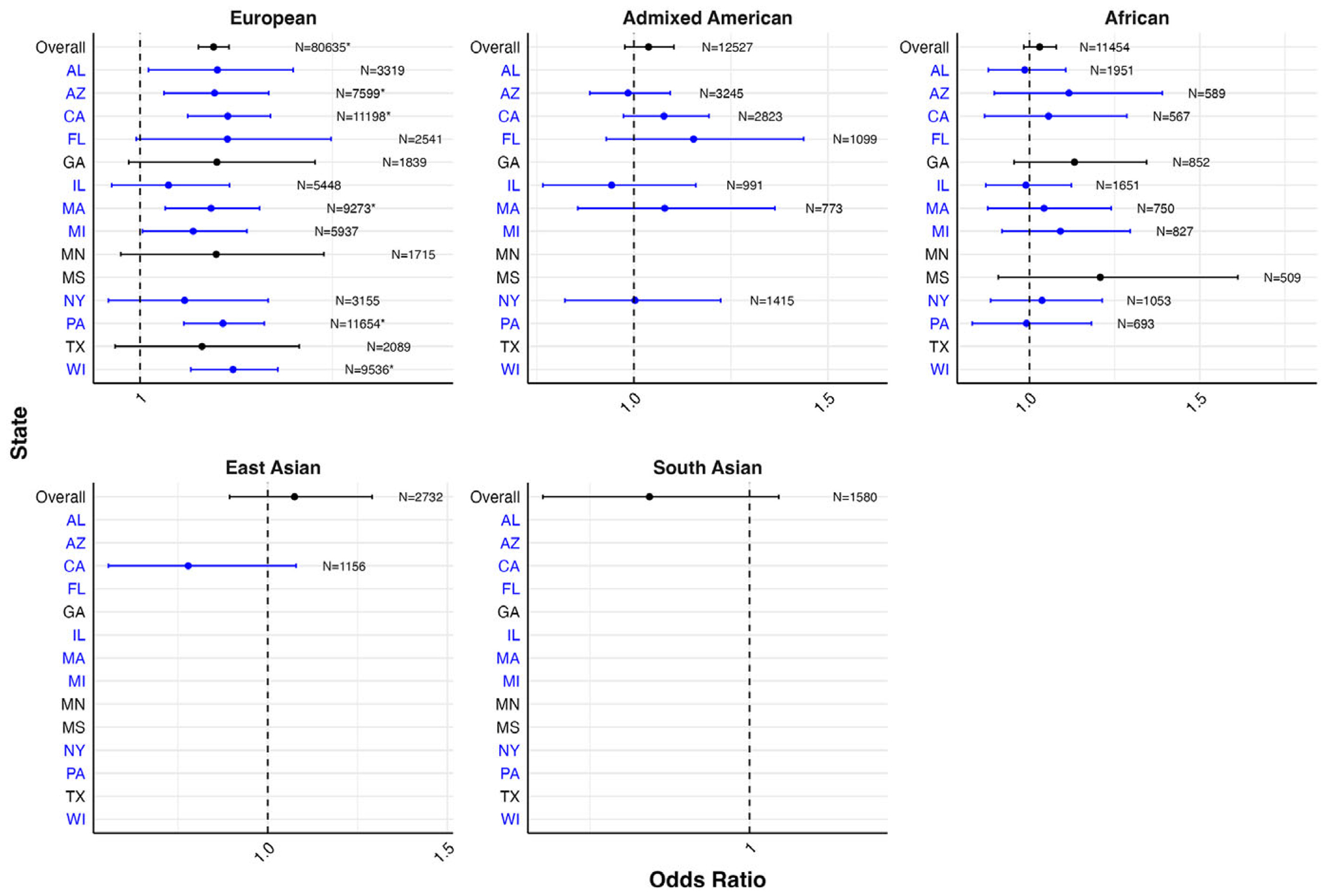
Forest plot of odds ratios and 95% confidence intervals for one standard deviation change in PRS with self-reported lifetime depression by genetic ancestry and state of residence in the PMH genomic subsample. *Note:* States and corresponding estimates in blue denote locations of *All of Us* enrollment centers (All of Us Research Program, 2024b). Models adjusted for 10 PCs. Models with sample sizes <500 overall and/or ≤5 for either response to a binary depression outcome were excluded from analyses in order to ensure statistical power and compliance with data dissemination policies. Estimates in subgroups with smaller sample sizes should be interpreted with caution. *Statistical significance after false discovery rate correction.

Figure 3 presents the PC-adjusted estimates for the associations between PRS and self-reported depression in the PMH sample, from models stratified by genetic ancestry and state of residence, with underlying estimates provided in Supplementary Table S6. The strongest magnitudes of associations were observed among participants of European genetic ancestry. Among European genetic ancestry participants, a statistically significant association was observed for the PRS with self-reported lifetime depression in five out of the 13 states evaluated, with the statistically significant associations ranging from 1.09 (95% CI: 1.03–1.16) in Massachusetts to 1.12 (95% CI: 1.07–1.18) in Wisconsin.

**Figure 4.**
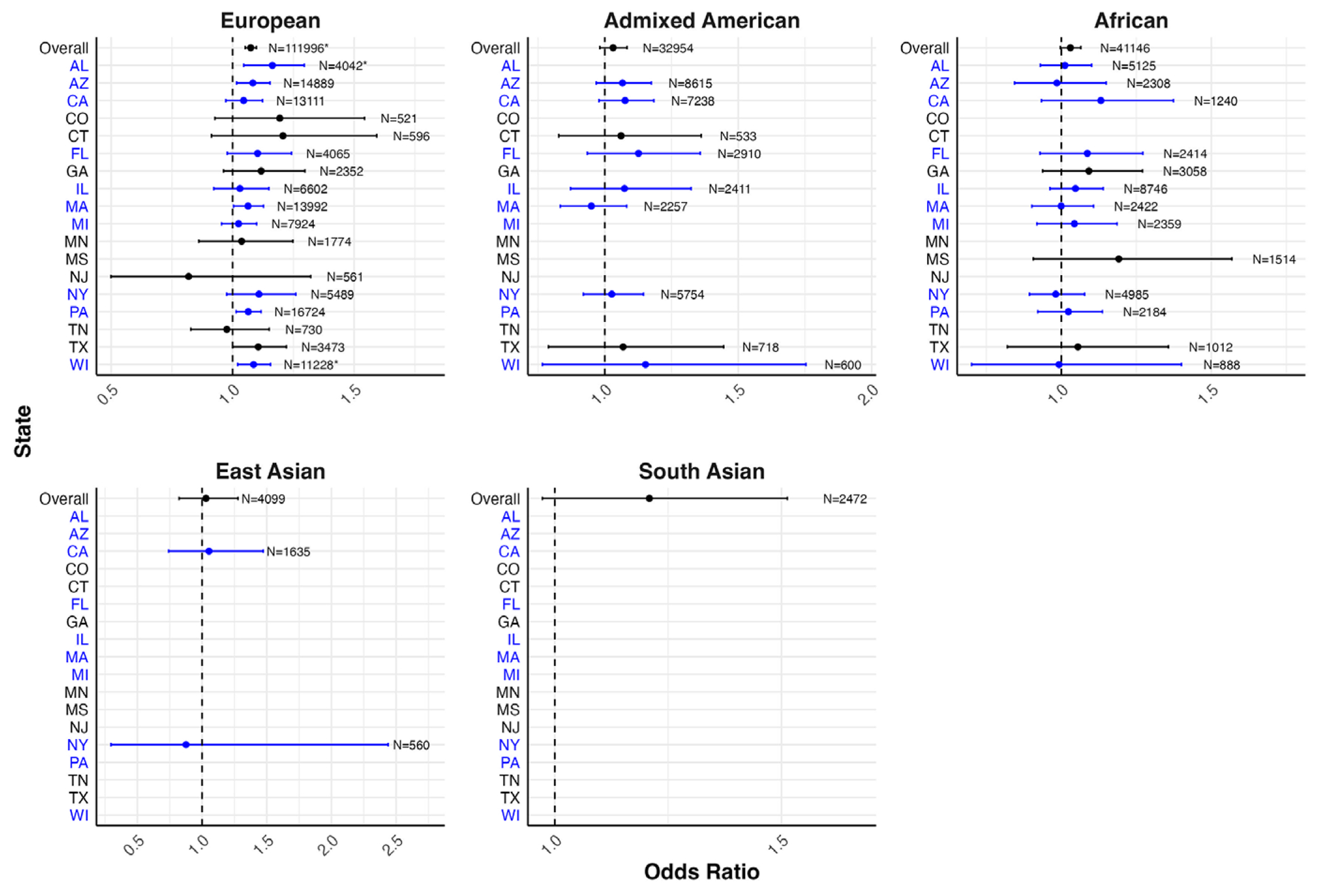
Forest plot of odds ratios and 95% confidence intervals for one standard deviation change in PRS on diagnosed lifetime depression by genetic ancestry and state of residence in the EHR genomic subsample. *Note:* States and corresponding estimates in blue denote locations of *All of Us* enrollment centers (All of Us Research Program, 2024b). Models adjusted for 10 PCs. Models with sample sizes <500 overall and/or ≤5 for either response to a binary depression outcome were excluded from analyses in order to ensure statistical power and compliance with data dissemination policies. Estimates in subgroups with smaller sample sizes should be interpreted with caution. *Statistical significance after false discovery rate correction.

Figure 4 presents the PC-adjusted estimates for the associations between PRS and diagnosed lifetime depression in the EHR sample, from models stratified by genetic ancestry and state of residence, with underlying estimates reported in Supplementary Table S6. The strongest association signals were also observed among participants of European genetic ancestry. Among European genetic ancestry participants, there was a statistically significant association between PRS and lifetime self-reported depression in two out of the 17 states evaluated, with the statistically significant associations ranging from 1.09 (95% CI: 1.02–1.16) in Wisconsin to 1.16 (95% CI: 1.05–1.29) in Alabama.

### Sensitivity analyses

We conducted sensitivity analyses using an expanded measure of diagnosed depression that additionally included non-major depression diagnostic codes. PC-adjusted effect estimates from sensitivity analyses for the overall and state-stratified EHR sample are presented in Supplementary Figure S2 and Supplementary Table S5. The findings were largely similar, although slightly attenuated, compared to those in the primary analyses. PC-adjusted effect estimates from sensitivity analyses stratified by genetic ancestry are presented in Supplementary Figure A3 and Supplementary Table A6, with findings again closely mirroring those in the primary analyses.

## Discussion

The present study has three main findings. First, there is substantial variation in recorded depression prevalence across U.S. states in *All of Us*, both in the PMH and the EHR samples. Second, the prevalence and observed correlates of lifetime depression in *All of Us* were dependent on the data source of the depression phenotype (i.e., whether self-reported from PMH or documented diagnosis from EHR data), underscoring that the choice of depression measurement may influence the results of studies leveraging the* All of Us* data platform. Third, despite relatively strong selection factors into the current genetic subsample, we found that associations of the PRS were largely stable across self-reported lifetime depression and diagnosed lifetime depression. Aside from outlier states of residence with relatively small sample sizes, observed statistically significant PRS associations were generally smaller than those in existing meta-analyses and recent studies (approximately 1.2–1.4) (Adams, 2024; Fanelli et al., 2022) which could in part be due to selection factors or measurement error.

Prevalence differences in depression by U.S. state found here may derive from two sources arising from the study design. First, observed differences in prevalence may reflect that participants enrolled into the *All of Us* Research Program are not randomly sampled or representative. For example, the highest prevalence of depression was observed in Tennessee, where *All of Us* recruitment was conducted at a healthcare provider organization predominately serving low-income communities where depression prevalence may be concentrated due to the impact of social determinants of health (Inokuchi, Mehta, & Burke, 2023). Low prevalence in some enrollment sites in *All of Us* did not provide mental health encounters as part of the submitted EHR, thus prevalence estimates in these sites are incomplete. Beyond sample selection and EHR linkage, prevalence variation may also reflect underlying state-level differences in depression prevalence due to differences in social and political contexts that impact occurrences of depression (Lee et al., 2023), or potential differences by state in willingness to report depressive symptoms or availability of services for depression (Cummings, Wen, Ko, & Druss, 2013). Data from the U.S. Behavioral Risk Factor Surveillance Survey (BRFSS) suggest that depression prevalence varies more than two-fold in magnitude across the U.S. (Lee, Wang, et al., 2023) states that emerged in the *All of Us* data as having high prevalence based on the PMH data, such as Mississippi and Tennessee, also have among the highest prevalence in the BRFSS data, suggesting consistency at least in rank order of depression capture in *All of Us*.

Both of these sources of variation are consequential for studies of risk factors, determinants, and outcomes. Prevalence differences impact not only statistical power but also the magnitude of associations generated from the data (Keyes et al., 2015). To understand why, consider a potential reason why the prevalence of depression differs by U.S. states: poverty status. Poverty is implicated in depression incidence and persistence (Kirkbride et al., 2024), and varies considerably across the United States according to economies, state-level benefit generosity, and many other factors (Bitler, Hoynes, & Kuku, 2017; Laird, Parolin, Waldfogel, & Wimer, 2018). If poverty, or any other factors that determine the magnitude of depression prevalence by state, modifies the effect of any risk factor assessed in relation to depression, then the magnitude of the association for that risk factor will vary (Ettman, Goicoechea, Stuart, & Dean, 2024; Keyes, Pakserian, Rudolph, Salum, & Stuart, 2024). This mathematical fact is well known in the epidemiological literature (Hernán & VanderWeele, 2011; Rudolph, Levy, Schmidt, Stuart, & Ahern, 2020) and is a principal reason why there is a growing recognition that target sample validity and target sample representativeness are critically important to generating actionable research findings in terms of public health or clinical practice (Fried, Flake, & Robinaugh, 2022; Westreich, Edwards, Lesko, Cole, & Stuart, 2019). Statistical approaches to addressing target validity and selection bias are an active area of research, and applying such approaches may be beneficial to studies using biobanks for etiological research (Lee, Wang, et al., 2023; Rudolph et al., 2020; Rudolph, Levy, & van der Laan, 2021).

Our results also showed variation in lifetime depression prevalence based on measurement technique, either by self-report or ascertainment from the medical record. Neither measurement technique in *All of Us* reflects gold standard approaches such as structured clinical interviews; thus, both sources likely reflect considerable measurement error (Glanville et al., 2021). EHR data likely underreport true prevalence, given that most individuals with depression do not utilize any services for their symptoms (Hom, Stanley, & Joiner, 2015; Wang et al., 2005). Combining data across measurement sources is complicated due to differing samples for inclusion, and time frames of potential diagnoses. As such, future research using *All of Us* and other large biobanks should consider which measurement source is most applicable to the study question, and for etiological research, reporting resources for multiple measurement sources as separate outcomes. Indeed, the minimal phenotyping approach used in *All of Us*, which relies on broad diagnostic categories rather than detailed clinical assessments, likely contributed to both the low concordance between measurement approaches and the attenuated PRS effect sizes compared to studies using more refined phenotypic definitions (Cai et al., 2020; Glanville et al., 2021). Binary depression measures may have introduced measurement error that reduced the precision of genetic associations, as previous research has demonstrated that more comprehensive phenotyping approaches yield stronger and more reliable PRS associations with psychiatric outcomes (Adams, 2024; Fanelli et al., 2022). In addition to the measurement error, differences in correlates between the measurement approaches underscores potential pathways to receiving a depression diagnosis. For example, in the EHR data, depression diagnoses were associated with older age, in contrast to self-reported depression. EHR data may thus reflect different pathways to care than self-reported diagnosis, and the phenotype itself thus represents both depression occurrence and depression identification. Previous studies indicate that signals for risk factors may be heightened as the number of different measures for which participants have positive depression data increases; thus, developing methods to increase sample coverage for multiple measurement sources, and aligning time frames of diagnosis, may be a promising way forward (Glanville et al., 2021).

We observed minimal, but present, variation in the magnitude of the odds ratio between the PRS and depression status, both across U.S. states and across measurement techniques. Observed magnitudes were generally lower than the bounds of what would be expected based on existing literature (Ettman et al., 2024; Mitchell et al., 2021), aside from some outlier states with generally small sample size and thus limited power to detect associations. Notably, despite the low kappa we found between PMH and EHR depression measures – indicating these methods identify substantially different individuals as cases – the PRS effect estimates remained relatively similar across these measurement approaches. This suggests that polygenic risk may be capturing a broader vulnerability to depression that transcends specific diagnostic contexts or measurement methods. Generally, the interpretation of this consistency is that the factors that determine prevalence differences across states likely do not interact with polygenic risk for depression. Put another way, social/structural and genetic determinants are both well documented to impact depression incidence and prevalence, but they do not substantially interact with each other in these data, at least when examining polygenic depression risk. This finding extends the broader literature on the gene–environment interaction in depression; whereas there has been substantial interest and intriguing preliminary findings, generally robust and replicable gene–environment interaction effects for depression have been few (Grillo, 2024). The similar PRS associations across divergent phenotyping approaches further suggests that genetic risk may operate through mechanisms that are detectable regardless of how depression is ascertained. Additional research into the specificity of the relationship between PRS and depression by examining the association between the PRS estimated here with other diagnosis such as bipolar and schizophrenia (Bulik-Sullivan et al., 2015; Lee et al., 2013) is an important next step to further elucidate the genetic underpinning of major psychiatric disorders.

Potential threats to validity when using *All of Us* should be carefully considered, based on the specific analyses and subgroups of interest. At present, the availability of genetic data is correlated both with having a depression diagnosis and with the demographic factors that themselves are deterministic of depression prevalence. This represents a selection bias, given that both the outcome and the potential predictors are deterministic of the availability of exposure data. The impact of this selection bias may ease as additional genetic data are added to *All of Us*, and more robust gene–environment findings may be detectable. Importantly, *All of Us* has recently released initial data from its Mental Health and Well-Being surveys which include much more extensive self-report measures of depression that may mitigate the limitations of minimal phenotyping noted here.

Limitations of the study should be noted. We relied on available *All of Us* data, which will continue to be updated in the future, thus these results may change as the data becomes more complete. The study is currently gathering survey data specifically designed to assess data on depression and other mental health phenotypes in more detail. Furthermore, we relied on available EHR linkages, but there are gaps in EHR coverage as not all sites linked participants to mental health diagnostic codes, thus ‘lifetime’ diagnoses in EHR are underrepresented. For some institutions, mental health encounters were systematically not shared as part of the EHR data submission to the program; thus, we have missing data on EHR mental health visits. Furthermore, participants who received healthcare at multiple institutions may not have all visits reflected in their EHR records, and the time coverage of mental health diagnoses in EHR systems may also vary by state; thus, there is additional potential for undercounting of depression diagnoses given that EHR systems may not cover the life course of all individuals in the sample. However, our goal in this paper was to highlight the ways in which data availability patterns may influence results, and the potential selection into the EHR dataset was of scientific interest. The available recorded depression diagnoses in *All of US* represent minimal phenotyping, which may lead to reduced signal for genetic effects as well as increased measurement error more generally (Cai et al., 2020). Thus, the completeness of data capture is an important limitation within the *All of Us* study to highlight for future analyses. Finally, we included the state of residence of the participant, but their state of residence and state of clinical visit may be different, which may have implications for interpreting whether prevalence differences arise due to state-level or clinic-level factors.

In conclusion, as genetic research in large biobanks like the *All of Us* Research Program continues to expand, careful consideration of sample composition, recruitment strategies, and measurement approaches is crucial for generating actionable findings for public health and clinical practice. Future studies should prioritize representative sampling and standardized measurement to enhance the validity and generalizability of genetic discoveries in psychiatric research.

## Supplementary Material

supplementary material

The supplementary material for this article can be found at http://doi.org/10.1017/S0033291725102420.

## Figures and Tables

**Table 1.** Demographic distribution of samples by depression diagnosis

	PMH sample					EHR sample				
	Lifetime depression	row %	No lifetime depression	Total	p	Lifetime depression	row %	No lifetime depression	Total	p
	48,497	26.2	136,735	185,232		32,480	11.3	254,535	287,015	

Age, years					<0.001					<0.001

<25	3,311	30.3	7,617	10,928		1,349	7.5	16,579	17,928	

25–54	24,062	30.3	55,442	79,504		14,490	11.1	115,751	130,241	

>54	21,124	22.3	73,676	94,800		16,641	12.0	122,204	138,845	

Gender					<0.001					<0.001

Female	34,736	29.7	82,386	117,122		21,931	12.8	149,503	171,434	

Male	11,471	18.5	50,561	62,032		9,576	8.8	98,633	108,209	

Other	566	51.3	537	1,103		225	18.2	1,008	1,233	

Missing or Skipped	1,635	33.5	3,251	4,886		748	12.2	5,391	6,139	

Race/ethnicity					<0.001					<0.001

Hispanic or Latino	4,467	20.9	16,922	21,389		5,604	10.4	48,451	54,055	

White	36,674	28.4	92,416	129,090		18,767	12.5	131,637	150,404	

Black or African American	3,289	19.4	13,629	16,918		5,702	10.0	51,475	57,177	

Asian or Native Hawaiian or Pacific Islander	897	14.5	5,300	6,197		421	5.1	7,914	8,335	

Middle Eastern or North African	169	17.6	793	962		150	9.3	1,455	1,605	

More than one population	1,040	31.2	2,290	3,330		530	12.0	3,886	4,416	

Missing or Skipped	1,961	26.7	5,385	7,346		1,306	11.8	9,717	11,023	

Survey language					<0.001					0.159

English	47,732	26.6	131,876	179,608		30,320	11.3	238,212	268,532	

Spanish	672	13.0	4,498	5,170		2,071	11.6	15,720	17,791	

English and Spanish	93	20.5	361	454		89	12.9	603	692	

Highest educational attainment					<0.001					<0.001

<High school graduate	1,158	19.4	4,818	5,976		3,285	11.8	24,583	27,868	

High school graduate or GED	4,768	24.3	14,886	19,654		6,786	12.0	49,992	56,778	

>High school graduate	41,257	26.7	113,402	154,659		21,410	11.1	171,776	193,186	

Missing or skipped	1,314	26.6	3,629	4,943		999	10.9	8,184	9,183	

Annual income					<0.001					<0.001

<$25 k	8,526	31.0	19,003	27,529		10,762	14.4	64,062	74,824	

$25 k–$50 k	8,887	30.6	20,158	29,045		5,481	12.9	37,038	42,519	

$50 k–$100 k	12,825	27.7	33,450	46,275		5,613	10.9	45,932	51,545	

>$100 k	13,226	22.8	44,819	58,045		4,258	7.4	52,947	57,205	

Missing or Skipped	5,033	20.7	19,305	24,338		6,366	10.4	54,556	60,922	

Genomics data					<0.001					<0.001

No	19,876	28.2	50,617	70,493		9,779	12.1	71,061	80,840	

Yes	28,621	24.9	86,118	114,739		22,701	11.0	183,474	206,175	

*Note:* p Values for chi-squared tests with continuity correction.
