# Supplementary material for: Using large biobanks for psychiatric genomic research: Consistency of clinical and genetic aspects of recorded depression across US states in the All of Us Research Program
Source: Psychol Med. Author manuscript; Available in PMC 2026 Mar 14. (PMC12885337; doi:10.1017/S0033291725102420)
Supplement: supplementary material [file NIHMS2144971-supplement-supplementary_material.docx]

**SUPPLEMENTARY FIGURES**

**Supplementary Figure 1.** Sample flow chart


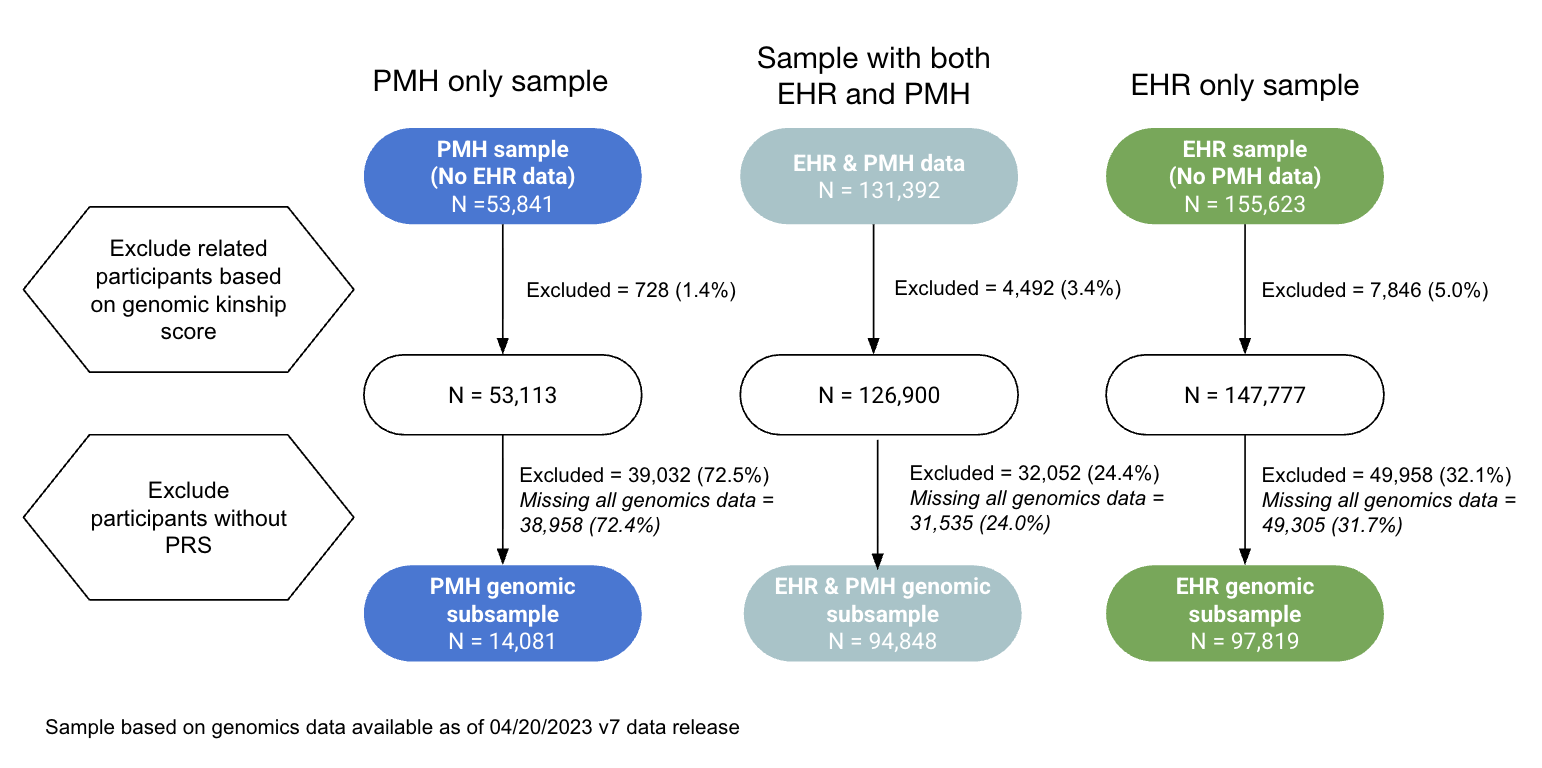


**Supplementary Figure 2.** Forest plot of logistic regression estimates of PRS on the expanded definition of lifetime depression stratified by genetic ancestry both overall and further stratified by state of residence in the EHR genomic subsample


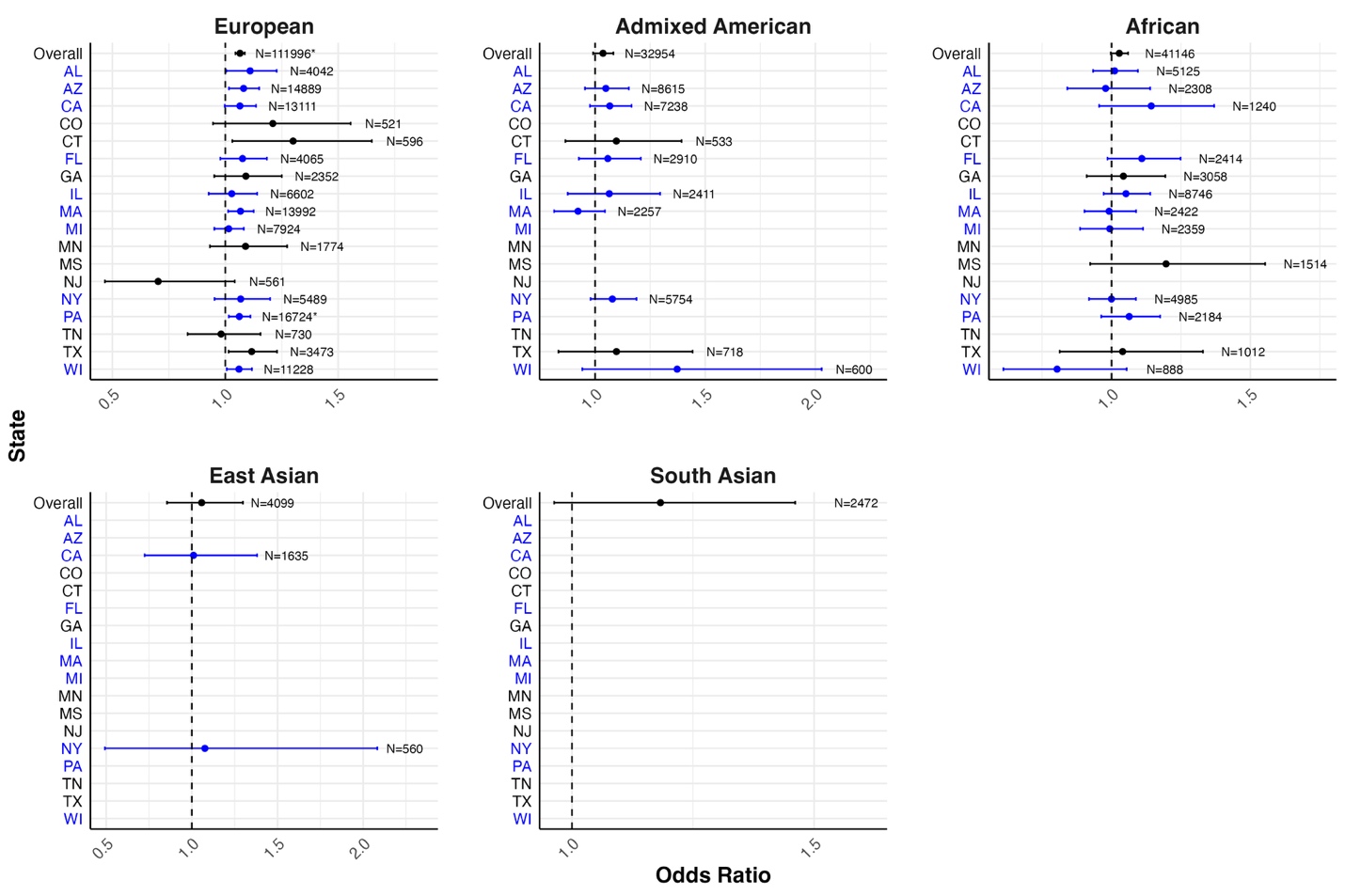


*Notes:* States and corresponding estimates in blue denote locations of All of Us enrollment centers.^19^ Models adjusted for 10 PCs. Models with sample sizes <500 overall and/or ≤ 5 for either response to a binary depression outcome were excluded from analyses in order to ensure statistical power and compliance with data dissemination policies. Estimates in subgroups with smaller sample sizes should be interpreted with caution.

* denotes statistical significance after false discovery rate correction.

**Supplementary Figure 3.** Forest plot of logistic regression estimates of PRS on the expanded definition of lifetime depression overall and stratified by state of residence in the EHR genomic subsample


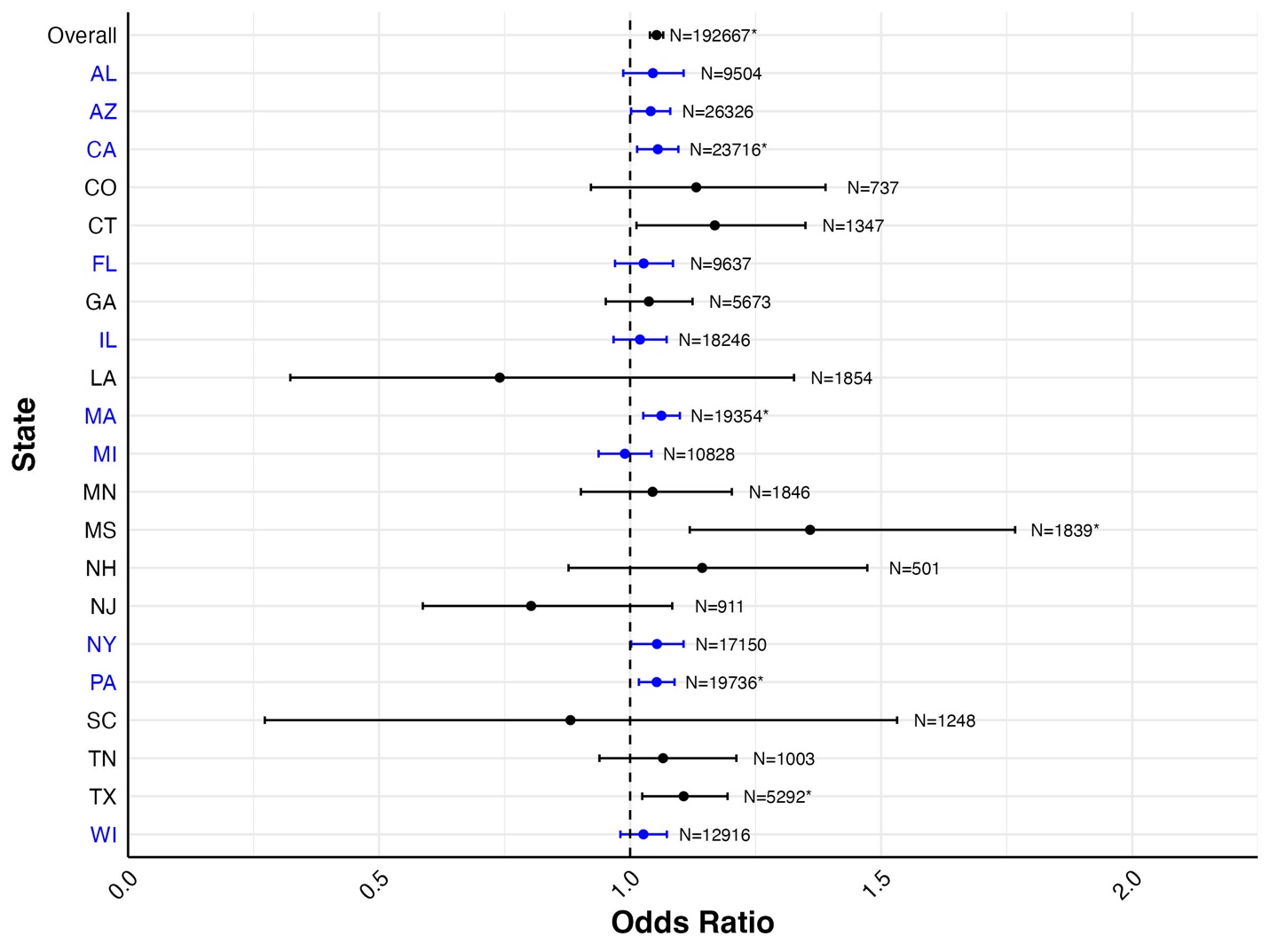


*Notes:* Models adjusted for 10 PCs.

* denotes statistical significance after false discovery rate correction.

**SUPPLEMENTARY TABLES**

**Supplementary Table 1.** ICD diagnostic codes used for EHR lifetime depression definitions

| **INCLUDED CODES** | | | |
| --- | --- | --- | --- |
| **Diagnosis** | **ICD era** | **ICD code** | **ICD description** |
| **Major depression** | 9 | 296.2 | Major depressive affective disorder, single episode, unspecified |
|  | 9 | 296.21 | Major depressive affective disorder, single episode, mild |
|  | 9 | 296.22 | Major depressive affective disorder, single episode, moderate |
|  | 9 | 296.23 | Major depressive affective disorder, single episode, severe |
|  | 9 | 296.25 | Major depressive affective disorder, single episode, in partial or unspecified remission |
|  | 9 | 296.26 | Major depressive affective disorder, single episode, in full remission |
|  | 9 | 296.3 | Major depressive affective disorder, recurrent episode, unspecified |
|  | 9 | 296.31 | Major depressive affective disorder, recurrent episode, mild |
|  | 9 | 296.32 | Major depressive affective disorder, recurrent episode, moderate |
|  | 9 | 296.33 | Major depressive affective disorder, recurrent episode, severe, without mention of psychotic behavior |
|  | 9 | 296.35 | Major depressive affective disorder, recurrent episode, in partial or unspecified remission |
|  | 9 | 296.36 | Major depressive affective disorder, recurrent episode, in full remission |
|  | 10 | F32.0 | Major depressive disorder, single episode, mild |
|  | 10 | F32.1 | Major depressive disorder, single episode, moderate |
|  | 10 | F32.2 | Major depressive disorder, single episode, severe without psychotic features |
|  | 10 | F32.4 | Major depressive disorder, single episode, in partial remission |
|  | 10 | F32.5 | Major depressive disorder, single episode, in full remission |
|  | 10 | F32.9 | Major depressive disorder, single episode, unspecified |
|  | 10 | F33.0 | Major depressive disorder, recurrent, mild |
|  | 10 | F33.1 | Major depressive disorder, recurrent, moderate |
|  | 10 | F33.2 | Major depressive disorder, recurrent severe without psychotic features |
|  | 10 | F33.40 | Major depressive disorder, recurrent, in remission, unspecified |
|  | 10 | F33.41 | Major depressive disorder, recurrent, in partial remission |
|  | 10 | F33.42 | Major depressive disorder, recurrent, in full remission |
|  | 10 | F33.9 | Major depressive disorder, recurrent, unspecified |
| **Depression with psychosis** | 9 | 296.34 | Major depressive affective disorder, recurrent episode, severe, specified as with psychotic behavior |
|  | 9 | 298 | Depressive type psychosis |
|  | 10 | F32.3 | Major depressive disorder, single episode, severe with psychotic features |
|  | 10 | F33.3 | Major depressive disorder, recurrent, severe with psychotic symptoms |
| **Non-major depression *(Sensitivity Analysis)*** | 9 | 311 | Depressive disorder, not elsewhere classified |
|  | 9 | 300.4 | Dysthymic disorder |
|  | 9 | 309.1 | Prolonged depressive reaction |
|  | 10 | F32.89 | Other specified depressive episodes |
|  | 10 | F33.8 | Other recurrent depressive disorders |
|  | 10 | F43.21 | Adjustment disorder with depressed mood |

**Supplementary Table 2.** Cohen’s kappas for concordance between self-reported depression in the PMH samples and both depression and the expanded sensitivity analysis definition of depression in the EHR samples

| **State** | **N** | **Primary Sample** | **Primary Sample: Sensitivity analysis** | **N** | **Genomic subsample** | **Genomic subsample: Sensitivity analysis** |
| --- | --- | --- | --- | --- | --- | --- |
|  |  | Kappa (95% CI) | |  | Kappa (95% CI) | |
| AL | 4652 | 0.26 (0.23, 0.29) | 0.28 (0.25, 0.31) | 3602 | 0.27 (0.23, 0.3) | 0.29 (0.25, 0.32) |
| AZ | 15491 | 0.23 (0.21, 0.24) | 0.23 (0.22, 0.25) | 10534 | 0.23 (0.21, 0.25) | 0.24 (0.22, 0.26) |
| CA | 16831 | 0.23 (0.22, 0.25) | 0.26 (0.24, 0.28) | 12292 | 0.24 (0.22, 0.26) | 0.27 (0.25, 0.29) |
| CO | 811 | 0.32 (0.24, 0.4) | 0.34 (0.27, 0.42) | - | - | - |
| CT | 1019 | 0.19 (0.13, 0.25) | 0.24 (0.17, 0.3) | 701 | 0.19 (0.11, 0.27) | 0.23 (0.15, 0.31) |
| FL | 4785 | 0.37 (0.34, 0.4) | 0.44 (0.41, 0.47) | 3635 | 0.38 (0.34, 0.41) | 0.44 (0.4, 0.47) |
| GA | 3661 | 0.31 (0.27, 0.34) | 0.34 (0.3, 0.37) | 2600 | 0.32 (0.28, 0.36) | 0.36 (0.31, 0.4) |
| IL | 10999 | 0.3 (0.27, 0.32) | 0.32 (0.3, 0.34) | 7671 | 0.29 (0.26, 0.32) | 0.31 (0.29, 0.34) |
| MA | 14288 | 0.35 (0.33, 0.37) | 0.36 (0.34, 0.38) | 10803 | 0.35 (0.33, 0.37) | 0.36 (0.34, 0.38) |
| MI | 9305 | 0.33 (0.31, 0.35) | 0.37 (0.35, 0.39) | 6836 | 0.33 (0.31, 0.36) | 0.37 (0.35, 0.4) |
| MN | 1783 | 0.15 (0.11, 0.19) | 0.16 (0.12, 0.21) | 1304 | 0.15 (0.1, 0.2) | 0.16 (0.11, 0.21) |
| MS | 958 | 0.23 (0.14, 0.31) | 0.23 (0.14, 0.31) | 591 | 0.2 (0.1, 0.31) | 0.22 (0.12, 0.33) |
| NJ | 556 | 0.1 (0.02, 0.18) | 0.13 (0.04, 0.22) | - | - | - |
| NY | 7420 | 0.23 (0.21, 0.26) | 0.25 (0.22, 0.27) | 5528 | 0.24 (0.21, 0.27) | 0.25 (0.22, 0.28) |
| PA | 16747 | 0.36 (0.34, 0.37) | 0.39 (0.38, 0.41) | 12217 | 0.36 (0.34, 0.38) | 0.4 (0.38, 0.42) |
| SC | 1126 | 0 (-0.02, 0.02) | 0 (-0.02, 0.02) | 732 | 0.01 (-0.02, 0.03) | 0 (-0.03, 0.03) |
| TN | 768 | 0.34 (0.28, 0.41) | 0.34 (0.28, 0.41) | 507 | 0.39 (0.31, 0.47) | 0.41 (0.33, 0.48) |
| TX | 2665 | 0.34 (0.3, 0.38) | 0.36 (0.32, 0.4) | 2062 | 0.33 (0.28, 0.38) | 0.35 (0.3, 0.4) |
| WI | 13840 | 0.29 (0.28, 0.31) | 0.37 (0.35, 0.39) | 9788 | 0.29 (0.27, 0.31) | 0.37 (0.35, 0.39) |

**Supplementary Table 3.** Demographic distribution of EHR sample by sensitivity analysis definition of depression

|  | **EHR Sample** | | | | |
| --- | --- | --- | --- | --- | --- |
|  | **Lifetime depression** | *row %* | **No lifetime depression** | **Total** | **p-value** |
|  | 40,027 | 13.9% | 246,988 | 287,015 |  |
| **Age** |  |  |  |  | *<0.001* |
| <25 | 1,525 | 8.5% | 16,403 | 17,928 |  |
| 25 - 54 | 17,357 | 13.3% | 112,884 | 130,241 |  |
| >54 | 21,145 | 15.2% | 117,700 | 138,845 |  |
| **Gender** |  |  |  |  | *<0.001* |
| Female | 27,018 | 15.8% | 144,416 | 171,434 |  |
| Male | 11,816 | 10.9% | 96,393 | 108,209 |  |
| Other | 257 | 20.8% | 976 | 1,233 |  |
| Missing or Skipped | 936 | 15.2% | 5,203 | 6,139 |  |
| **Race / Ethnicity** |  |  |  |  | *<0.001* |
| Hispanic or Latino | 7,034 | 13.0% | 47,021 | 54,055 |  |
| White | 23,150 | 15.4% | 127,254 | 150,404 |  |
| Black or African American | 6,936 | 12.1% | 50,241 | 57,177 |  |
| Asian or Native Hawaiian or Pacific Islander | 500 | 6.0% | 7,835 | 8,335 |  |
| Middle Eastern or North African | 180 | 11.2% | 1,425 | 1,605 |  |
| More than one population | 614 | 13.9% | 3,802 | 4,416 |  |
| Missing or Skipped | 1,613 | 14.6% | 9,410 | 11,023 |  |
| **Survey language** |  |  |  |  | *<0.001* |
| English | 37,190 | 13.8% | 231,342 | 268,532 |  |
| Spanish | 2,720 | 15.3% | 15,071 | 17,791 |  |
| English & Spanish | 117 | 16.9% | 575 | 692 |  |
| **Highest educational attainment** |  |  |  |  | *<0.001* |
| < High School Graduate | 4,010 | 14.4% | 23,858 | 27,868 |  |
| High School Graduate or GED | 8,209 | 14.5% | 48,569 | 56,778 |  |
| > High School Graduate | 26,535 | 13.7% | 166,651 | 193,186 |  |
| Missing or Skipped | 1,273 | 13.9% | 7,910 | 9,183 |  |
| **Annual Income** |  |  |  |  | *<0.001* |
| <$25k | 12,671 | 16.9% | 62,153 | 74,824 |  |
| $25k - $50k | 6,729 | 15.8% | 35,790 | 42,519 |  |
| $50k - $100k | 7,089 | 13.8% | 44,456 | 51,545 |  |
| >$100k | 5,396 | 9.5% | 51,647 | 57,043 |  |
| Missing or Skipped | 7,980 | 13.1% | 52,942 | 60,922 |  |
| **Genomics data** |  |  |  |  | *<0.001* |
| No | 11,933 | 14.8% | 68,907 | 80,840 |  |
| Yes | 28,094 | 13.6% | 178,081 | 206,175 |  |
|  |  |  |  |  |  |

*Notes:* P-values for chi-squared tests with continuity correction.

**Supplementary Table 4.** Demographic distribution of samples by genomics data indicator

|  | **PMH Sample** | | | | |  | **EHR Sample** | | | | |
| --- | --- | --- | --- | --- | --- | --- | --- | --- | --- | --- | --- |
|  | **Genomics data** | *row %* | **No genomics data** | **Total** | **p-value** |  | **Genomics data** | *row %* | **No genomics data** | **Total** | **p-value** |
|  | 114,739 | 61.9% | 70,493 | 185,232 |  |  | 206,175 | 71.8% | 80,840 | 287,015 |  |
| **Age** |  |  |  |  | *<0.001* |  |  |  |  |  | *<0.001* |
| <25 | 6,514 | 59.6% | 4,414 | 10,928 |  |  | 12,522 | 69.8% | 5,406 | 17,928 |  |
| 25 - 54 | 47,734 | 60.0% | 31,770 | 79,504 |  |  | 93,137 | 71.5% | 37,104 | 130,241 |  |
| >54 | 60,491 | 63.8% | 34,309 | 94,800 |  |  | 100,516 | 72.4% | 38,329 | 138,845 |  |
| **Gender** |  |  |  |  | *<0.001* |  |  |  |  |  | *0.012* |
| Female | 72,922 | 62.3% | 44,200 | 117,122 |  |  | 123,278 | 71.9% | 48,156 | 171,434 |  |
| Male | 38,431 | 62.0% | 23,601 | 62,032 |  |  | 77,566 | 71.7% | 30,643 | 108,209 |  |
| Other | 598 | 50.2% | 594 | 1,192 |  |  | 850 | 68.9% | 383 | 1,233 |  |
| Missing or Skipped | 2,788 | 57.1% | 2,098 | 4,886 |  |  | 4,481 | 73.0% | 1,658 | 6,139 |  |
| **Race / Ethnicity** |  |  |  |  | *<0.001* |  |  |  |  |  | *<0.001* |
| Hispanic or Latino | 14,117 | 66.0% | 7,272 | 21,389 |  |  | 38,159 | 70.6% | 15,896 | 54,055 |  |
| White | 78,972 | 61.2% | 50,118 | 129,090 |  |  | 110,044 | 73.2% | 40,360 | 150,404 |  |
| Black or African American | 10,872 | 64.3% | 6,046 | 16,918 |  |  | 40,074 | 70.1% | 17,103 | 57,177 |  |
| Asian or Native Hawaiian or Pacific Islander | 3,761 | 60.7% | 2,436 | 6,197 |  |  | 5,786 | 69.4% | 2,549 | 8,335 |  |
| Middle Eastern or North African | 623 | 64.8% | 339 | 962 |  |  | 1,084 | 67.5% | 521 | 1,605 |  |
| More than one population | 1,979 | 59.4% | 1,351 | 3,330 |  |  | 3,138 | 71.1% | 1,278 | 4,416 |  |
| Missing or Skipped | 4,415 | 60.1% | 2,931 | 7,346 |  |  | 7,890 | 71.6% | 3,133 | 11,023 |  |
| **Survey language** |  |  |  |  | *<0.001* |  |  |  |  |  | *0.063* |
| English | 110,772 | 61.7% | 68,836 | 179,608 |  |  | 192,759 | 71.8% | 75,773 | 268,532 |  |
| Spanish | 3,652 | 70.6% | 1,518 | 5,170 |  |  | 12,915 | 72.6% | 4,876 | 17,791 |  |
| English & Spanish | 315 | 69.4% | 139 | 454 |  |  | 501 | 72.4% | 191 | 692 |  |
| **Highest educational attainment** |  |  |  |  | *<0.001* |  |  |  |  |  | *<0.001* |
| < High School Graduate | 3,883 | 65.0% | 2,093 | 5,976 |  |  | 19,402 | 69.6% | 8,466 | 27,868 |  |
| High School Graduate or GED | 12,434 | 63.3% | 7,220 | 19,654 |  |  | 40,048 | 70.5% | 16,730 | 56,778 |  |
| > High School Graduate | 95,512 | 61.8% | 59,147 | 154,659 |  |  | 140,284 | 72.6% | 52,902 | 193,186 |  |
| Missing or Skipped | 2,910 | 58.9% | 2,033 | 4,943 |  |  | 6,441 | 70.1% | 2,742 | 9,183 |  |
| **Annual Income** |  |  |  |  | *<0.001* |  |  |  |  |  | *<0.001* |
| <$25k | 17,330 | 63.0% | 10,199 | 27,529 |  |  | 52,535 | 70.2% | 22,289 | 74,824 |  |
| $25k - $50k | 17,882 | 61.6% | 11,163 | 29,045 |  |  | 30,570 | 71.9% | 11,949 | 42,519 |  |
| $50k - $100k | 28,477 | 61.5% | 17,798 | 46,275 |  |  | 37,779 | 73.3% | 13,766 | 51,545 |  |
| >$100k | 35,557 | 61.3% | 22,488 | 58,045 |  |  | 42,577 | 74.4% | 14,628 | 57,205 |  |
| Missing or Skipped | 15,493 | 63.7% | 8,845 | 24,338 |  |  | 42,714 | 70.1% | 18,208 | 60,922 |  |
| **Lifetime depression** |  |  |  |  | *<0.001* |  |  |  |  |  | *<0.001* |
| No | 86,118 | 63.0% | 50,617 | 136,735 |  |  | 183,474 | 72.1% | 71,061 | 254,535 |  |
| Yes | 28,621 | 59.0% | 19,876 | 48,497 |  |  | 22,701 | 69.9% | 9,779 | 32,480 |  |
| **EHR sensitivity analysis depression** |  |  |  |  |  |  |  |  |  |  | *<0.001* |
| No |  |  |  |  |  |  | 178,081 | 72.1% | 68,907 | 246,988 |  |
| Yes |  |  |  |  |  |  | 28,094 | 70.2% | 11,933 | 40,027 |  |
|  |  |  |  |  |  |  |  |  |  |  |  |

*Notes:* P-values for chi-squared tests with continuity correction.

**Supplementary Table 5.** Odds ratios from logistic regression models estimating the association between PRS and depression outcomes overall and stratified by state in both the PMH and EHR genomic subsamples

|  |  |  |  |  |  |  |
| --- | --- | --- | --- | --- | --- | --- |
|  | **PMH Genomic subsample** | | **EHR Genomic subsample** | | **EHR Genomic subsample: Sensitivity analysis** | |
| **State** | *N* | *OR (95% CI)* | *N* | *OR (95% CI)* | *N* | *OR (95% CI)* |
| Overall | 108,928 | 1.05 (1.04, 1.07)* | 192,667 | 1.05 (1.04, 1.07)* | 192,667 | 1.05 (1.04, 1.07)* |
| AL | 5,557 | 1.07 (1.01, 1.14) | 9,504 | 1.06 (1, 1.13) | 9,504 | 1.05 (0.99, 1.11) |
| AZ | 11,697 | 1.07 (1.03, 1.12)* | 26,326 | 1.04 (1, 1.08) | 26,326 | 1.04 (1, 1.08) |
| CA | 16,099 | 1.09 (1.05, 1.13)* | 23,716 | 1.05 (1, 1.09) | 23,716 | 1.06 (1.01, 1.1)* |
| CO | - | - | 737 | 1.12 (0.91, 1.38) | 737 | 1.13 (0.92, 1.39) |
| CT | 727 | 1.15 (0.95, 1.39) | 1,347 | 1.08 (0.93, 1.26) | 1,347 | 1.17 (1.01, 1.35) |
| FL | 4,228 | 1.02 (0.94, 1.1) | 9,637 | 0.99 (0.92, 1.06) | 9,637 | 1.03 (0.97, 1.09) |
| GA | 2,871 | 1.07 (0.98, 1.16) | 5,673 | 1.07 (0.98, 1.17) | 5,673 | 1.04 (0.95, 1.12) |
| IL | 8,436 | 1 (0.95, 1.05) | 18,246 | 1.02 (0.96, 1.08) | 18,246 | 1.02 (0.97, 1.07) |
| LA | 545 | 1.05 (0.86, 1.26) | 1,854 | 0.73 (0.31, 1.34) | 1,854 | 0.74 (0.32, 1.33) |
| MA | 11,258 | 1.06 (1.02, 1.1)* | 19,354 | 1.07 (1.03, 1.1)* | 19,354 | 1.06 (1.03, 1.1)* |
| MI | 7,064 | 1.02 (0.97, 1.08) | 10,828 | 1.01 (0.95, 1.07) | 10,828 | 0.99 (0.94, 1.04) |
| MN | 1,778 | 1.08 (0.97, 1.2) | 1,846 | 1 (0.84, 1.19) | 1,846 | 1.04 (0.9, 1.2) |
| MS | 674 | 1.15 (0.94, 1.4) | 1,839 | 1.45 (1.15, 1.99)* | 1,839 | 1.36 (1.12, 1.77)* |
| NH | - | - | 501 | 1.13 (0.84, 1.49) | 501 | 1.14 (0.88, 1.47) |
| NJ | 533 | 1.09 (0.87, 1.35) | 911 | 0.92 (0.64, 1.31) | 911 | 0.8 (0.59, 1.08) |
| NY | 6,074 | 1.03 (0.96, 1.1) | 17,150 | 1.03 (0.98, 1.09) | 17,150 | 1.05 (1, 1.11) |
| PA | 12,895 | 1.07 (1.03, 1.11)* | 19,736 | 1.06 (1.02, 1.09)* | 19,736 | 1.05 (1.02, 1.09) |
| SC | 752 | 1.1 (0.88, 1.38) | 1,248 | 0.82 (0.22, 1.55) | 1,248 | 0.88 (0.27, 1.53) |
| TN | 594 | 0.97 (0.82, 1.15) | 1,003 | 1.07 (0.94, 1.21) | 1,003 | 1.07 (0.94, 1.21) |
| TX | 2,708 | 1.07 (0.98, 1.16) | 5,292 | 1.11 (1.02, 1.2)* | 5,292 | 1.11 (1.02, 1.19)* |
| WA | 522 | 1.05 (0.87, 1.27) | - | - | - | - |
| WI | 10,284 | 1.11 (1.06, 1.16)* | 12,916 | 1.06 (1.01, 1.12) | 12,916 | 1.03 (0.98, 1.07) |
|  |  |  |  |  |  |  |

*Notes:* Models adjusted for 10 PCs.

* denotes statistical significance after false discovery rate correction.

**Supplementary Table 6.** Odds ratios from logistic regression models estimating the association between PRS and depression outcomes stratified by ancestry and state in both the PMH and EHR genomic subsamples

|  |  |  |  |  |  |  |  |
| --- | --- | --- | --- | --- | --- | --- | --- |
|  |  | **PMH Genomic subsample** | | **EHR Genomic subsample** | | **EHR Genomic subsample: Sensitivity analysis** | |
| **Ancestry** | **State** | *N* | *OR (95% CI)* | *N* | *OR (95% CI)* | *N* | *OR (95% CI)* |
| African | **Overall** | 11,454 | 1.03 (0.98, 1.08) | 41,146 | 1.03 (1, 1.06) | 41,146 | 1.03 (1, 1.06) |
|  | AL | 1,951 | 0.99 (0.88, 1.11) | 5,125 | 1.01 (0.93, 1.1) | 5,125 | 1.01 (0.93, 1.09) |
|  | AZ | 589 | 1.12 (0.9, 1.39) | 2,308 | 0.99 (0.84, 1.15) | 2,308 | 0.98 (0.84, 1.14) |
|  | CA | 567 | 1.06 (0.87, 1.29) | 1,240 | 1.13 (0.93, 1.37) | 1,240 | 1.14 (0.96, 1.37) |
|  | CO | - | - | - | - | - | - |
|  | CT | - | - | - | - | - | - |
|  | FL | - | - | 2,414 | 1.09 (0.93, 1.27) | 2,414 | 1.11 (0.99, 1.25) |
|  | GA | 852 | 1.13 (0.95, 1.34) | 3,058 | 1.09 (0.94, 1.27) | 3,058 | 1.04 (0.91, 1.19) |
|  | IL | 1,651 | 0.99 (0.87, 1.12) | 8,746 | 1.05 (0.96, 1.14) | 8,746 | 1.05 (0.97, 1.14) |
|  | LA | - | - | - | - | - | - |
|  | MA | 750 | 1.04 (0.88, 1.24) | 2,422 | 1 (0.9, 1.11) | 2,422 | 0.99 (0.9, 1.09) |
|  | MI | 827 | 1.09 (0.92, 1.3) | 2,359 | 1.04 (0.92, 1.19) | 2,359 | 0.99 (0.89, 1.11) |
|  | MN | - | - | - | - | - | - |
|  | MS | 509 | 1.21 (0.91, 1.61) | 1,514 | 1.19 (0.91, 1.57) | 1,514 | 1.2 (0.92, 1.55) |
|  | NJ | - | - | - | - | - | - |
|  | NY | 1,053 | 1.04 (0.89, 1.21) | 4,985 | 0.98 (0.89, 1.08) | 4,985 | 1 (0.92, 1.09) |
|  | PA | 693 | 0.99 (0.83, 1.18) | 2,184 | 1.02 (0.92, 1.14) | 2,184 | 1.06 (0.96, 1.18) |
|  | SC | - | - | - | - | - | - |
|  | TN | - | - | - | - | - | - |
|  | TX | - | - | 1,012 | 1.06 (0.82, 1.36) | 1,012 | 1.04 (0.81, 1.33) |
|  | WI | - | - | 888 | 0.99 (0.7, 1.4) | 888 | 0.8 (0.61, 1.05) |
| Admixed American | **Overall** | 12,527 | 1.04 (0.98, 1.1) | 32,954 | 1.04 (1, 1.07) | 32,954 | 1.03 (1, 1.06) |
|  | AL | - | - | - | - | - | - |
|  | AZ | 3,245 | 0.98 (0.89, 1.09) | 8,615 | 1.07 (0.97, 1.17) | 8,615 | 1.05 (0.95, 1.15) |
|  | CA | 2,823 | 1.08 (0.97, 1.19) | 7,238 | 1.08 (0.98, 1.18) | 7,238 | 1.07 (0.98, 1.17) |
|  | CO | - | - | - | - | - | - |
|  | CT | - | - | 533 | 1.06 (0.83, 1.36) | 533 | 1.1 (0.87, 1.39) |
|  | FL | 1,099 | 1.15 (0.93, 1.44) | 2,910 | 1.13 (0.93, 1.36) | 2,910 | 1.06 (0.93, 1.21) |
|  | GA | - | - | - | - | - | - |
|  | IL | 991 | 0.94 (0.77, 1.16) | 2,411 | 1.07 (0.87, 1.32) | 2,411 | 1.06 (0.88, 1.3) |
|  | LA | - | - | - | - | - | - |
|  | MA | 773 | 1.08 (0.86, 1.36) | 2,257 | 0.95 (0.83, 1.08) | 2,257 | 0.92 (0.81, 1.04) |
|  | MI | - | - | - | - | - | - |
|  | MN | - | - | - | - | - | - |
|  | MS | - | - | - | - | - | - |
|  | NJ | - | - | - | - | - | - |
|  | NY | 1,415 | 1 (0.82, 1.22) | 5,754 | 1.03 (0.92, 1.14) | 5,754 | 1.08 (0.98, 1.19) |
|  | PA | - | - | - | - | - | - |
|  | SC | - | - | - | - | - | - |
|  | TN | - | - | - | - | - | - |
|  | TX | - | - | 718 | 1.07 (0.79, 1.45) | 718 | 1.1 (0.83, 1.44) |
|  | WI | - | - | 600 | 1.15 (0.77, 1.75) | 600 | 1.37 (0.94, 2.03) |
| East Asian | **Overall** | 2,732 | 1.07 (0.89, 1.29) | 4,099 | 1.03 (0.82, 1.28) | 4,099 | 1.06 (0.85, 1.31) |
|  | AL | - | - | - | - | - | - |
|  | AZ | - | - | - | - | - | - |
|  | CA | 1,156 | 0.78 (0.56, 1.08) | 1,635 | 1.05 (0.74, 1.47) | 1,635 | 1.01 (0.72, 1.38) |
|  | CO | - | - | - | - | - | - |
|  | CT | - | - | - | - | - | - |
|  | FL | - | - | - | - | - | - |
|  | GA | - | - | - | - | - | - |
|  | IL | - | - | - | - | - | - |
|  | LA | - | - | - | - | - | - |
|  | MA | - | - | - | - | - | - |
|  | MI | - | - | - | - | - | - |
|  | MN | - | - | - | - | - | - |
|  | MS | - | - | - | - | - | - |
|  | NJ | - | - | - | - | - | - |
|  | NY | - | - | 560 | 0.88 (0.3, 2.44) | 560 | 1.08 (0.49, 2.08) |
|  | PA | - | - | - | - | - | - |
|  | SC | - | - | - | - | - | - |
|  | TN | - | - | - | - | - | - |
|  | TX | - | - | - | - | - | - |
|  | WI | - | - | - | - | - | - |
| European | **Overall** | 80,635 | 1.1 (1.08, 1.12)* | 111,996 | 1.07 (1.05, 1.1)* | 111,996 | 1.06 (1.04, 1.09)* |
|  | AL | 3,319 | 1.1 (1.01, 1.2) | 4,042 | 1.16 (1.05, 1.29)* | 4,042 | 1.11 (1, 1.23) |
|  | AZ | 7,599 | 1.1 (1.03, 1.17)* | 14,889 | 1.08 (1.02, 1.15) | 14,889 | 1.08 (1.02, 1.15) |
|  | CA | 11,198 | 1.11 (1.06, 1.17)* | 13,111 | 1.05 (0.97, 1.12) | 13,111 | 1.06 (1, 1.14) |
|  | CO | - | - | 521 | 1.19 (0.93, 1.54) | 521 | 1.21 (0.95, 1.56) |
|  | CT | - | - | 596 | 1.21 (0.91, 1.59) | 596 | 1.3 (1.03, 1.65) |
|  | FL | 2,541 | 1.11 (1, 1.25) | 4,065 | 1.1 (0.98, 1.24) | 4,065 | 1.08 (0.98, 1.18) |
|  | GA | 1,839 | 1.1 (0.99, 1.23) | 2,352 | 1.12 (0.96, 1.3) | 2,352 | 1.09 (0.95, 1.25) |
|  | IL | 5,448 | 1.04 (0.96, 1.12) | 6,602 | 1.03 (0.92, 1.15) | 6,602 | 1.03 (0.93, 1.14) |
|  | LA | - | - | - | - | - | - |
|  | MA | 9,273 | 1.09 (1.03, 1.16)* | 13,992 | 1.06 (1, 1.13) | 13,992 | 1.07 (1.01, 1.13) |
|  | MI | 5,937 | 1.07 (1, 1.14) | 7,924 | 1.02 (0.95, 1.1) | 7,924 | 1.01 (0.95, 1.08) |
|  | MN | 1715 | 1.1 (0.97, 1.24) | 1,774 | 1.04 (0.86, 1.25) | 1,774 | 1.09 (0.93, 1.27) |
|  | MS | - | - | - | - | - | - |
|  | NJ | - | - | 561 | 0.82 (0.5, 1.32) | 561 | 0.7 (0.47, 1.04) |
|  | NY | 3,155 | 1.06 (0.96, 1.17) | 5,489 | 1.11 (0.98, 1.26) | 5,489 | 1.07 (0.95, 1.2) |
|  | PA | 11,654 | 1.11 (1.06, 1.16)* | 16,724 | 1.06 (1.01, 1.12) | 16,724 | 1.06 (1.02, 1.11)* |
|  | SC | - | - | - | - | - | - |
|  | TN | - | - | 730 | 0.98 (0.83, 1.15) | 730 | 0.98 (0.83, 1.16) |
|  | TX | 2,089 | 1.08 (0.97, 1.21) | 3,473 | 1.11 (1, 1.22) | 3,473 | 1.12 (1.01, 1.23) |
|  | WI | 9,536 | 1.12 (1.07, 1.18)* | 11,228 | 1.09 (1.02, 1.16)* | 11,228 | 1.06 (1.01, 1.12) |
| South Asian | **Overall** | 1,580 | 0.84 (0.68, 1.05) | 2,472 | 1.21 (0.97, 1.51) | 2,472 | 1.18 (0.96, 1.46) |
|  | AL | - | - | - | - | - | - |
|  | AZ | - | - | - | - | - | - |
|  | CA | - | - | - | - | - | - |
|  | CO | - | - | - | - | - | - |
|  | CT | - | - | - | - | - | - |
|  | FL | - | - | - | - | - | - |
|  | GA | - | - | - | - | - | - |
|  | IL | - | - | - | - | - | - |
|  | LA | - | - | - | - | - | - |
|  | MA | - | - | - | - | - | - |
|  | MI | - | - | - | - | - | - |
|  | MN | - | - | - | - | - | - |
|  | MS | - | - | - | - | - | - |
|  | NJ | - | - | - | - | - | - |
|  | NY | - | - | - | - | - | - |
|  | PA | - | - | - | - | - | - |
|  | SC | - | - | - | - | - | - |
|  | TN | - | - | - | - | - | - |
|  | TX | - | - | - | - | - | - |
|  | WI | - | - | - | - | - | - |
|  |  |  |  |  |  |  |  |

*Notes:* Models adjusted for 10 PCs. Models with sample sizes <500 overall and/or ≤ 5 for either response to a binary depression outcome were excluded from analyses in order to ensure statistical power and compliance with data dissemination policies. Estimates in subgroups with smaller sample sizes should be interpreted with caution.

* denotes statistical significance after false discovery rate correction.
